# Scale‐dependence in elk habitat selection for a reintroduced population in Wisconsin, USA

**DOI:** 10.1002/ece3.70346

**Published:** 2024-10-01

**Authors:** Jennifer L. Merems, Anna L. Brose, Jennifer Price Tack, Shawn Crimmins, Timothy R. Van Deelen

**Affiliations:** ^1^ Department of Forest and Wildlife Ecology, College of Agriculture and Life Sciences University of Wisconsin‐Madison Madison Wisconsin USA; ^2^ Wisconsin Department of Natural Resources Rhinelander Wisconsin USA; ^3^ US Geological Survey, Alaska Cooperative Fish and Wildlife Research Unit University of Alaska‐Fairbanks Fairbanks Alaska USA

**Keywords:** antipredator behavior, behavioral plasticity, ecological tradeoff, elk, habitat selection, limiting factors, scale‐dependent, ungulate

## Abstract

Habitat selection is a critical aspect of a species' ecology, requiring complex decision‐making that is both hierarchical and scale‐dependent, since factors that influence selection may be nested or unequal across scales. Elk (*Cervus canadensis*) ranged widely across diverse ecoregions in North America prior to European settlement and subsequent eastern extirpation. Most habitat selection studies have occurred within their contemporary western range, even after eastern reintroductions began. As habitat selection can vary by geographic location, available cover, season, and diel period, it is important to understand how a non‐migratory, reintroduced population in northern Wisconsin, USA, is limited by the lack of variation in topography, elevation, and vegetation. We tested scale‐dependent habitat selection on 79 adult elk from 2017 to 2020 using resource selection functions across temporal (i.e., seasonal) and spatial scales (i.e., landscape and home range). We found that selection varied both spatially and temporally, and elk selected areas with the greatest potential to influence fitness at larger scales, meaning elk selected areas closer to escape cover and further from “risky” features (e.g., annual wolf territory centers, county roads, and highways). We found stronger avoidance of annual wolf territory centers during spring, suggesting elk were selecting safer habitats during calving season. Elk selected habitats with less canopy cover across both spatial scales and all seasons, suggesting that elk selected areas with better access to forage as early seral stage stands have greater forage biomass than closed‐canopy forests and direct solar radiation to provide warmth in the cooler seasons. This study provides insight into the complexity of hierarchical decision‐making, such as how risky habitat features and land cover type influence habitat selection differently across seasons and spatial scales, influencing the decision‐making of elk. Scale‐dependent behavior is crucial to understand within specific geographic regions, as these decisions scale up to influence population dynamics.

## Graphical abstract

Habitat selection is a critical aspect of a species' ecology requiring complex decision‐making that is both hierarchical and scale‐dependent, since factors that influence selection may be nested or unequal across scales. This study investigates how risky features at different spatial and temporal scales influence species' decision making surrounding life history characteristics. It provides insight into the complexity of making informed decisions in which an individual is responding to their immediate environment while simultaneously making decisions in context of the larger landscape. Scale‐dependent behavior is crucial to understand within specific geographic regions as these decisions scale up to influence population dynamics.

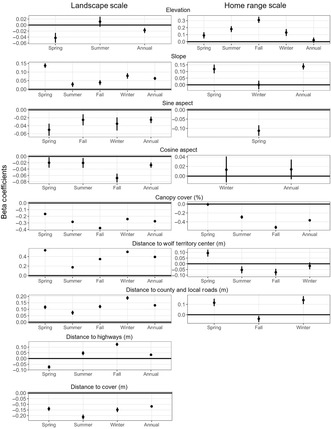

## 1 INTRODUCTION

Habitat selection is a critical aspect of a species' ecology (Rosenzweig, 1981) requiring complex decision‐making that is both hierarchical and scale‐dependent, since factors that influence selection may be nested or unequal across scales (Anderson, Turner, et al., 2005; Boyce et al., 2003; Johnson et al., 2002; Kie et al., 2002; Senft et al., 1987; Zweifel‐Schielly et al., 2009). For instance, one limiting factor at large scales (e.g., landscape scale (i.e., 2nd order; Johnson, 1980), annual) may determine behavior at successively finer scales until, at a certain scale (e.g., home range scale (i.e., 3rd order; Johnson, 1980), daily), a different limiting factor becomes more important for increasing fitness (i.e., when selection decisions at the larger scale (2nd order; Johnson, 1980) become sufficient enough to overcome the initial limiting factor) and animals thus may need to alter their selection decisions (Basille et al., 2013; Hebblewhite & Merrill, 2009; Rettie & Messier, 2000). Animals respond to their immediate environment while simultaneously making decisions in the context of the larger landscape (Boyce et al., 2003; Ciarniello et al., 2007; Johnson et al., 2004). One hypothesis is that factors crucial to individual fitness are more likely to operate at the landscape scale (Dussault et al., 2004; Rettie & Messier, 2000; Senft et al., 1987), such as abiotic factors like topography, which can impact forage quality and quantity (Mysterud et al., 2001), and snow cover, which can restrict access to foraging (Bailey et al., 1996; Boyce et al., 2003; Senft et al., 1987). Therefore, individuals must develop a system of scale‐dependent spatial behavior strategies that optimizes access to nutritious forage (Senft et al., 1987), as spatial and temporal variation influences forage quality and quantity, particularly in temperate climates with intense snowfall (Zweifel‐Schielly et al., 2009), while buffering against environmental variation (e.g., change patterns of habitat use for thermoregulation, optimal foraging, etc.; Senft et al., 1987; Huey et al., 2003; Kearney et al., 2009; Zweifel‐Schielly et al., 2009; Eisenberg et al., 2014).

Habitat selection is defined as disproportionate use of resources relative to what is available (Johnson, 1980; Mayor et al., 2009) and is often scale‐dependent (Witt et al., 2012). Selection is influenced by spatial scales (e.g., bite size, home range (i.e., 3rd order; Johnson, 1980), and landscape scales (i.e., 2nd order; Johnson, 1980; Senft et al., 1987; Bailey et al., 1996)) and temporal scales (e.g., seasonal dynamics of food availability (Nielsen et al., 2003), diel variation (Roberts et al., 2017), and past experiences (Merkle et al., 2014)). Spatial and temporal scales are both equally important, as they are linked, both conceptually and practically (e.g., landscape characteristics such as edge habitats have greater influence on elk [*Cervus canadensis*] selection during summer and winter at the landscape scale compared to other seasons and scales; Wiens, 1989; Bissonette, 1996; Jones & Hudson, 2002; Boyce et al., 2003). Understanding scale‐dependent habitat selection is crucial because scale‐dependent behavior ultimately influences population dynamics (Anderson, Turner, et al., 2005; Pulliam & Danielson, 1991).

Habitat selection in ungulates is strongly influenced by terrain features (Boyce et al., 2003), climatic conditions (Dussault et al., 2004), forage distribution (Fryxell et al., 2004), predation risk (Mech, 1977), and competition (Fretwell & Lucas, 1970). Predation risk can arise from landscape features associated with an increased probability of mortality (Hebblewhite et al., 2005; Kittle et al., 2008). For example, small areas of shared use (i.e., limited number of patches with high resource availability, watering holes; Sih, 2005, Smith et al., 2019), may increase the spatial and temporal predictability of ungulate presence, increasing their susceptibility to a mortality event (Suraci et al., 2022). Risky habitat features can influence the perception of risk, which forces behavioral modification of ungulates (i.e., non‐consumptive or risk effects; Huggler et al., 2022), resulting in ungulates using areas with increased forest cover which reduces risk but increases energetic costs as they forage more as biomass is less in these areas (Anderson, Forester, et al., 2005).

Elk (*Cervus canadensis*) ranged widely across diverse ecoregions in North America prior to European settlement but were extirpated from their eastern range by the 1870s (O'Gara & Dundas, 2002; Popp et al., 2014). Consequently, most studies of elk habitat selection have occurred within their contemporary western range, even after reintroduction of populations east of the Mississippi River (hereafter, eastern elk) began in the early 20th century (Amor et al., 2019; Anderson, Turner, et al., 2005; Boyce et al., 2003; O'Gara & Dundas, 2002). Understanding habitat selection at the population level is important because selection can vary by geographic location, available cover, season, and diel period (e.g., distance to water being important at certain times of day; Ager et al., 2003; Godvik et al., 2009; Roberts et al., 2017). Therefore, it is critical to understand habitat selection across eastern elk populations to make informed management decisions because populations in eastern North America occur in substantially different ecosystems relative to populations in western North America (Amor et al., 2019; Anderson, Turner, et al., 2005).

For example, some eastern populations live in landscapes with vast open areas (>200 ha; Kentucky, Tennessee, Virginia) creating prime foraging habitats (Quinlan et al., 2022) while others rely on small forest openings (<50 ha; Missouri and Wisconsin; Smith et al., 2018, Brose, 2021). Additionally, non‐migratory populations in the Great Lakes region are limited by lack of variation in topography and vegetation (Gilbert et al., 2010; Hinton et al., 2020; Smith et al., 2018), while western populations experience greater diversity in topography, allowing both resident and migratory populations to capitalize on seasonal differences in resource availability (Fryxell et al., 1988). Mortality factors also varied in source and magnitude. The largest mortality factors in eastern populations varied across populations but encompass harvest/poaching, disease, vehicle collision, predation, and accidents/other (Popp et al., 2014), while for western populations mortality consistently comes from harvest (the largest source), unknown causes, gray wolves (*Canis lupus*), and mountain lions (*Puma concolor*; Brodie et al., 2013). Wisconsin is the only eastern population where wolves are their primary predator and largest source of mortality (Merems, 2023).

Our objective was to understand habitat selection of a reintroduced population of elk in northern Wisconsin across two spatial scales (home range and landscape) and four seasons (spring, summer, fall, and winter). We predict that 1) elk will show spatiotemporally dependent selection for environmental factors across spatial scales and seasons and 2) strength of selection (use relative to availability) for environmental factors will be more strongly correlated to factors that influence survival (i.e., avoiding risky areas such as roads and wolf territories and selecting areas with less canopy cover; Anderson, Turner, et al., 2005; Cook et al., 2016) at the largest spatial scale (i.e., landscape scale).

## 2 MATERIALS AND METHODS

### 2.1 Study area

The Northern Elk Range (NER) is in the Chequamegon‐Nicolet National Forest and Flambeau River State Forest located in Wisconsin, USA. The NER is approximately 4200 km^2^, primarily forested (>65%), and comprised dominantly of publicly owned lands (68%). Forested areas are comprised of coniferous (dominated by red and white pine [*Pinus resinosa* and *P. stroubus*], and balsam fir [*Abies balsamea*]), deciduous (dominated by sugar maple [*Acer saccharum*], quaking aspen [*Populus tremuloides*], yellow and paper birch [*Betula alleghaniensis* and *B. papyrifera*]), and mixed forests. The rest of the landscape is a mosaic of wetland communities. Gray wolves and black bears (*Ursus americanus*) inhabit this study area and prey on elk. Other prominent carnivores are coyotes (*Canis latrans*), bobcats (*Lynx rufus*) and red foxes (*Vulpes vulpes*; Anderson, Turner, et al., 2005). Additionally, white‐tailed deer (*Odocoileus virginianus*) are abundant throughout this range and the primary prey species for gray wolves along with American beavers (*Castor canadensis*), snowshoe hares (*Lepus americanus*), and other small mammals (Thompson, 1952).

Topography within the range consists of flat to rolling terrain, soils derived from silt‐covered and sandy loam glacial tills, with elevations ranging between 260 and 595 m (Wisconsin Department of Natural Resources, 2015). The climate is cool and moist, with average annual temperature range from −14.7°C to 26.1°C, annual precipitation reaching 76–83 cm, and annual snow depths ≥58 cm (Wisconsin Department of Natural Resources, 2015). During the study period (2017–2020), the mean monthly temperature in spring (Mar–May), summer (Jun–Aug), fall (Sep–Nov), and winter (Dec–Feb) were Hi 9.3°C Lo 0°C, Hi 24.7°C Lo 13.7°C, Hi 11.3°C Lo 4°C, and Hi −5.3°C Lo −13°C, respectively. Mean monthly precipitation amounts for spring, summer, fall, winter were 89 mm ± 33.2, 118 mm ± 3.6, 79.7 mm ± 20.6, and 35 mm ± 15.9, respectively. Average cumulative hours of sun during spring, summer, fall, and winter were 124 ± 45.2, 215.6 ± 18, 153.7 ± 40.6, and 52.3 ± 12, respectively (weatherworld.com). Mean growing season is 115 days, making it the shortest growing season of all landscapes in Wisconsin (Wisconsin Department of Natural Resources, 2015).

### 2.2 Location data

Adult elk (male and female) were captured by the Wisconsin Department of Natural Resources (WDNR) during winter using portable corral traps and were fitted with global positioning system (GPS) collars (Vectronics Aerospace, Berlin, Germany; Lotek Wireless Inc., Seattle, Washington) between 2017 and 2020 that were programmed to record locations at 13‐h intervals (WDNR, 2024). We filtered locations for positional accuracy (fixes removed with dilution of precision (DOP) > 5 for 2‐dimensional fixes and DOP >10 for 3‐dimensional fixes; Lewis et al., 2007). Individuals with <15 fixes per season were removed from the study (Anderson, Turner, et al., 2005).

### 2.3 Resource selection function modeling framework

**FIGURE 1:**
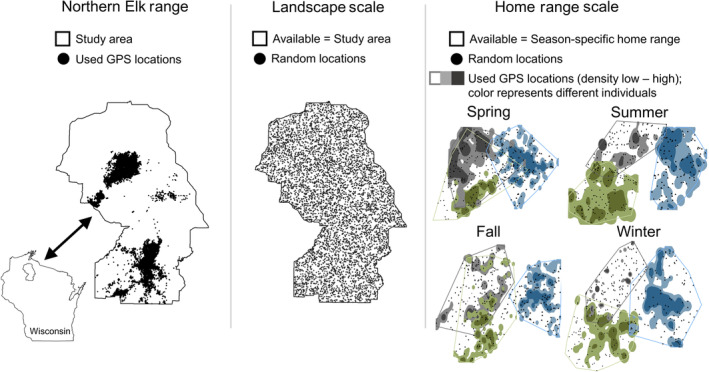
Example schematic of the sampling design for modeling habitat selection across two spatial scales and four seasons for elk in the Northern Elk Range in Wisconsin, USA. Used GPS locations represent all individuals included in the study; landscape scale random locations represent locations for one individual; and the home range scale represents three individuals.

We sought to understand resource selection patterns of elk relative to the availability of resources in the NER, as we were interested in determining the extent of suitable habitat within this range along with scale‐dependent differences in resource selection. We considered two spatial scales of selection: landscape scale (population‐level selection of home ranges within a region) and the home range scale (individual‐level selection within home ranges within the landscape; Spitz et al., 2019). To quantify the landscape scale, we treated the areas within the NER boundary as available locations and GPS locations of individual elk as used locations (Figure 1). To quantify the home range scale, we treated the area within an individual's annual home range as available and known GPS locations as used (Figure 1). Home range boundaries were estimated using minimum convex polygons (MCPs) with a convex hull from ArcGIS (ESRI, 2012). For both scales, used and available locations were specific for an individual elk; with 6000 available locations per individual at the landscape scale and 100 available locations per individual at the home range scale (determined using the methods of Long et al., 2014). All available locations were randomly distributed in either the study area or within individual home range boundaries. We then considered temporal estimates across spatial scales and GPS locations pooled across years (2017–2020) within biologically meaningful seasons: spring (March 1–May 31), summer (June 1–August 31), fall (September 1–November 30), and winter (December 1–February 28/29; Hinton et al., 2020).

We estimated resource selection functions using a use‐availability design where used locations (coded 1) and available locations (coded 0; Long et al., 2014) were used to quantify available habitat. We fit generalized linear mixed models using the lme4 package in R (Bates et al., 2015) with a logit link function and a binomial error distribution to used and available elk locations (Gillies et al., 2006; Long et al., 2014; Table 1), including a random intercept grouped by individual to account for autocorrelation and unequal sample sizes among individuals, and a random intercept grouped by year to account for annual variation (Zuur et al., 2009). We evaluated predictive strength of each model using *k*‐fold cross validation with ten iterations (Boyce et al., 2003). Each model was trained on 80% of the dataset (comprising 80% of the animals), with the remaining 20% reserved for testing. We grouped random locations from each iteration of the test data into 10 equal bins based on the predicted probability of use, as calculated from the training data. We evaluated the models by comparing the median predicted value of each bin to the actual number of used locations within each bin, using the Spearman‐rank correlation. We averaged Spearman‐rank correlation coefficients (*r*_*s*_) across all 10 iterations to assess the predictive strength of each model (Boyce et al., 2003).

**TABLE 1:** Generalized linear equations used for each spatial and temporal model and their associated Spearman‐rank correlation coefficients (*r*_*s*_) and *p*‐value.

Scale	Model	*r*_*s*_	*p*‐value
Landscape	Used ~ fixed effects + (1|individual) + (1|year), All GPS locations	0.770	.059
	Used ~ fixed effects + (1|individual) + (1|year), Spring GPS locations	0.727	.042
	Used ~ fixed effects + (1|individual) + (1|year), Summer GPS locations	0.849	.003
	Used ~ fixed effects + (1|individual) + (1|year), Fall GPS locations	0.783	.010
	Used ~ fixed effects + (1|individual) + (1|year), Winter GPS locations	0.828	.008
Home range	Used ~ fixed effects^1^ + (1|individual) + (1|year), All GPS locations	0.461	.287
	Used ~ fixed effects^1^ + (1|individual) + (1|year), Spring GPS locations	−0.052	.404
	Used ~ fixed effects^1^ + (1|individual) + (1|year), Summer GPS locations	0.185	.366
	Used ~ fixed effects^1^ + (1|individual) + (1|year), Fall GPS locations	0.398	.326
	Used ~ fixed effects^1^ + (1|individual) + (1|year), Winter GPS locations	0.431	.148

*Note*: Fixed effects: Elevation + Slope + Cosine aspect + Sine aspect + Canopy + Distance to cover (≥ 40% canopy cover) + Distance to county roads + Distance to highways + Distance to wolf territory centers + Land cover type. Fixed effects^1^: Elevation + Slope + Cosine aspect + Sine aspect + Canopy + Distance to county roads + Distance to wolf territory centers + Land cover type.

At each spatial scale (landscape, home‐range), we built five different models: one for each of the four seasons (spring, summer, fall, winter) and one annual model in which we used data from all four seasons.

### 2.4 Resource variables

We selected habitat variables known to be important predictors of elk space use (Johnson et al., 2000; Long et al., 2014): (1) land cover type; (2) aspect; (3) slope; (4) elevation; (5) distance to public roads (county, local, highway); (6) distance to wolf territory centers; (7) percent canopy cover and (8) distance to cover (≥40% canopy cover). Land cover type was separated into 13 different categories at 30‐m resolution in the NER; deciduous forests, coniferous forests, crop rotations, developed‐high intensity, developed‐low intensity, emergent/wet meadows, floating aquatic herbaceous vegetation, forage grasslands, forested wetlands, idle grasslands, lowland scrub/shrub, mixed deciduous/coniferous forests, open water, and barren (the reference category; WiscLand 2‐Level 2, WDNR GIS Open Data Portal, n.d.). Slope, elevation, and aspect (a circular land‐surface parameter meaning a transformation to a continuous gradient of sine and cosine is required representing north–south or east–west gradients; Olaya, 2009) were derived from a 30‐m resolution digital elevation model (DEM; WDNR GIS Open Data Portal) using the Calculate Slope and Derive Aspect functions from ArcGIS (ESRI, 2012).

Within the NER there are five state highways and numerous county and local roadways throughout. We calculated ‘distance to road’ using the spatial layers Major Roads and County and Local Roads from the OpenStreetMap roads data for Wisconsin (WDNR GIS Open Data Portal). We measured distance by calculating the shortest distance between each 30‐pixel grid cell and the nearest road, separated by type. As many county and local roadways within the NER are unlabeled, we combined these into a general ‘county roads’ category.

One novel covariate we included was distance to wolf territory center (km). Using wolf territory data provided by the WDNR, which was shown to be an influential variable in early models of Wisconsin elk resource selection (Anderson, Turner, et al., 2005). We used the “Find Centroids” tool in ArcGIS to estimate the centers of wolf territory polygon data provided by the WDNR and measured distance as described prior. The WDNR used territory mapping to determine the location of all wolf territories for both collared and uncollared wolves annually (Fuller et al., 2002; Wydeven et al., 2009). Live‐trapping and radio collaring of wolves in Wisconsin began in 1979 and year‐round radiotracking enabled the WDNR to determine annual pack territories using ≥20 radio locations to estimate home ranges using MCPs (Mohr, 1947; Wydeven et al., 2009). For non‐collared packs, polygons were created around all locations of sign and tracks (collected during summer howl surveys, winter snow track surveys, location of dead wolves, and depredation investigations), and reports of observations of wolves within assumed packs (Wydeven et al., 2009). If packs were collared in the past, they were assumed to have the same territory area unless field sign indicated the area had shifted (Wydeven et al., 2009). Due to shifts in annual territories, wolf territory centers and their associated distance were measured each year (2017–2020), during which wolf territories encompassed the entire NER. MCPs used in this research were created by the WDNR.

We determined canopy cover (%) using the community tree canopy raster layer with 30‐m resolution (WDNR GIS Open Data Portal). We estimated distance to cover (≥40% canopy cover; Johnson et al., 2000) based on elk selecting sites closer to edges for security (Rowland et al., 2018) and to minimize travel time to find micro‐climates within cover and more nutritious forage in open areas (Skovlin et al., 2002). All covariates were measured at a 30‐m resolution and were checked for collinearity (covariates with |*r*| > 0.45 were removed from the model) at each of the spatial and temporal scales to be considered for the model. To facilitate model fitting and the direct comparison of model coefficients, we standardized all continuous covariates by subtracting the mean and dividing by the standard deviation (Gelman, 2008).

## 3 RESULTS

At both the landscape and home range scales, our annual models consisted of 32, 56, 79, and 64 GPS collared elk during 2017, 2018, 2019, and 2020, respectively. The total number of GPS locations collected during this time was 72,965. Our seasonal models consisted of 49, 64, and 61 elk during spring in 2018, 2019, and 2020, respectively (2017 was not included due to 0 individuals), totaling 19,412 GPS locations; 28, 45, 74, and 59 elk during summer totaling 17,798 GPS locations; 24, 42, 72, and 50 elk during fall totaling 18,443 GPS locations; and 28, 56, 73, and 56 elk during winter totaling 17,312 GPS locations. There were no collinearity issues between covariates at the landscape scale, but at the home range scale, distance to highways and distance to cover was removed across all models due to collinearity (Table 1). The landscape scale annual model yielded higher predictive accuracy compared to the home range scale annual model (landscape scale *r*_*s*_ = 0.770, *p*‐value = .059; home range scale *r*_*s*_ = 0.461, *p*‐value = .287; Table 1). Similarly, across seasons the landscape scale models exhibiting higher predictive performance compared to the home range scale models (landscape scale ranged from *r*_*s*_ = 0.727–849; home range scale ranged from *r*_*s*_ = −0.052–0.431; Table 1).

### 3.1 Landscape scale (2nd order selection): Annual model

**FIGURE 2:**
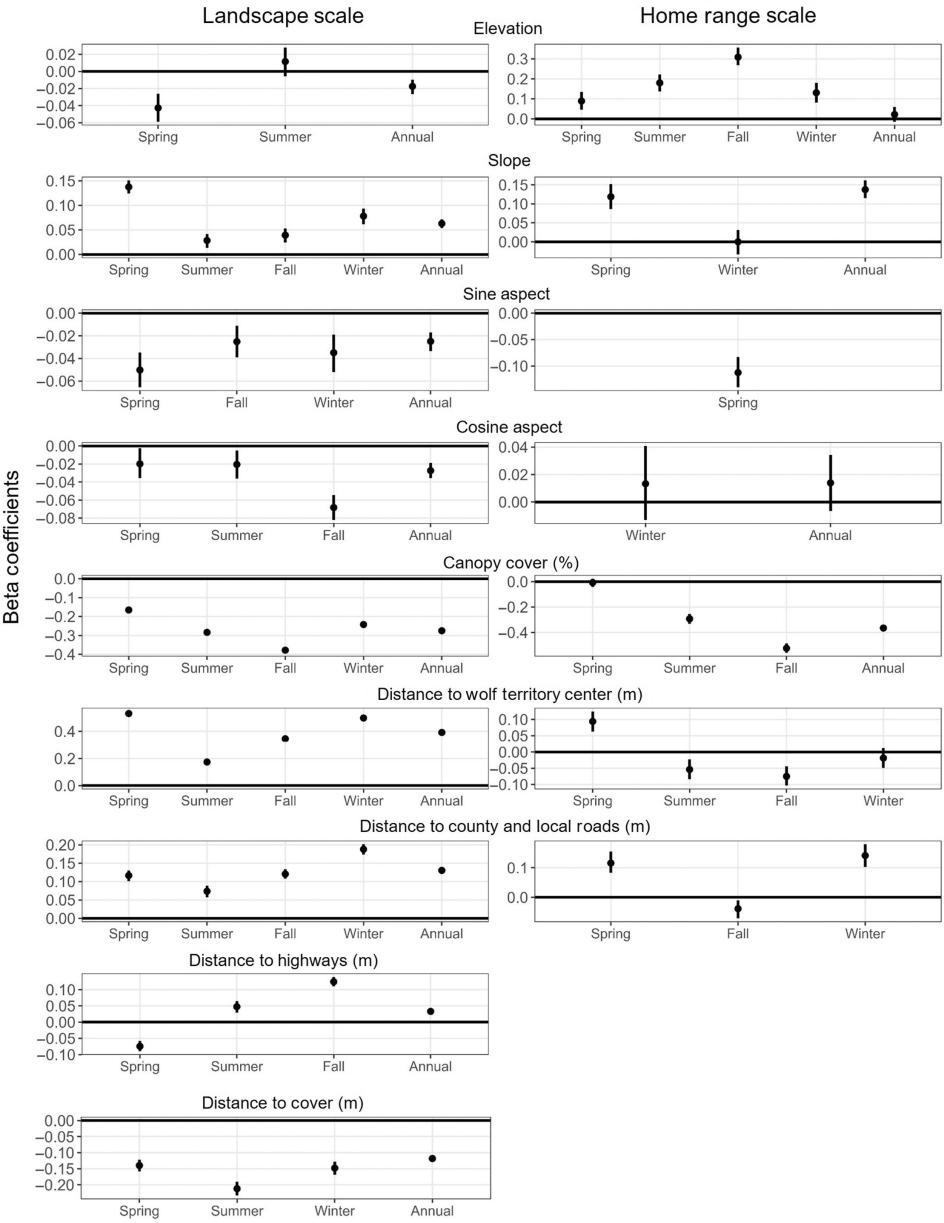
Scaled beta coefficients of landscape variables for resource selection by elk in the Northern Elk Range across two spatial scales and across four seasons in Wisconsin, USA; non‐significant covariates with *p*‐value >.05 were omitted and the horizontal line at *y* = 0 represents *β* = 0.

**FIGURE 3:**
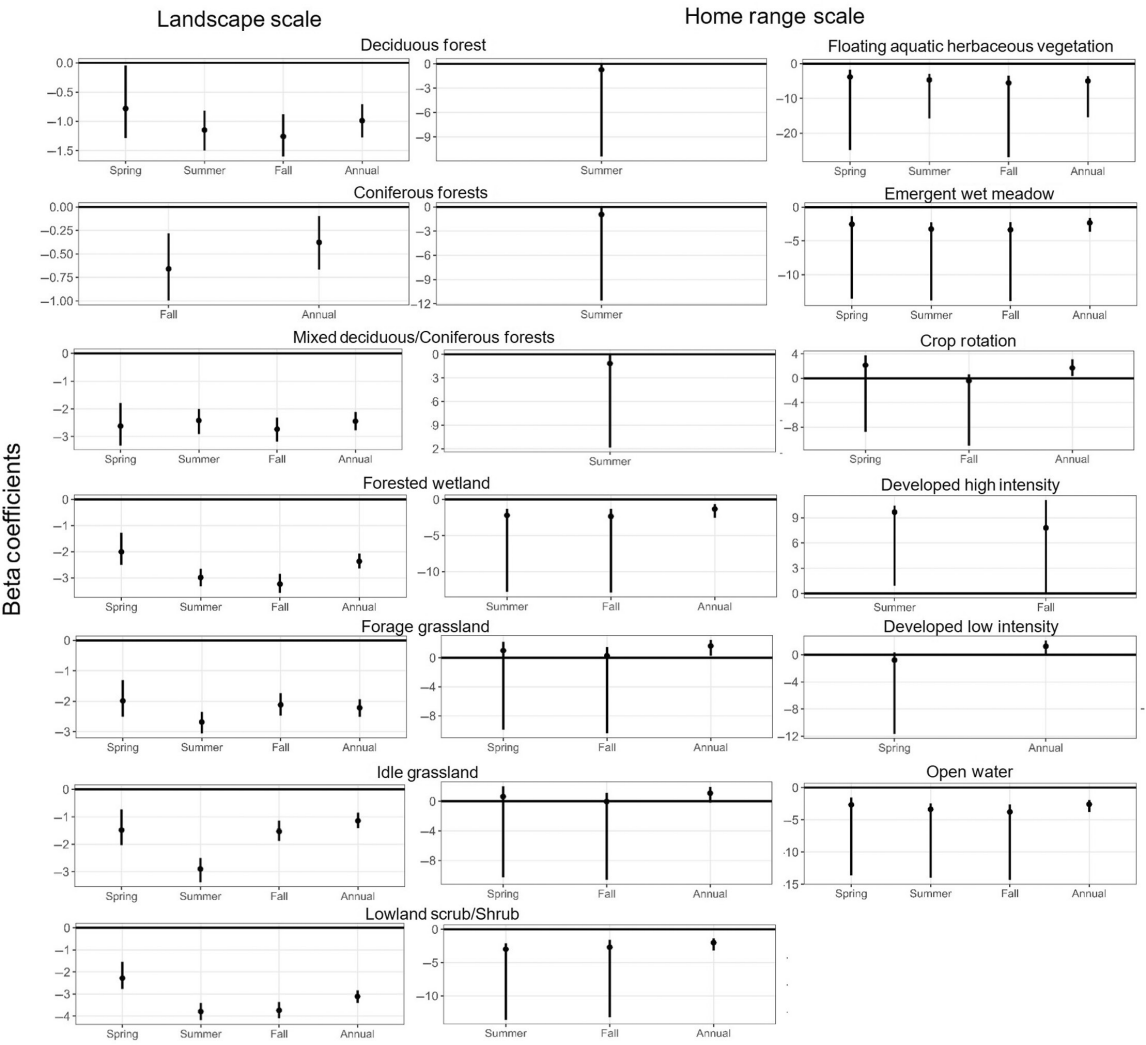
Scaled beta coefficients of land cover variables for resource selection by elk in the Northern Elk Range across two spatial scales and across four seasons in Wisconsin, USA; non‐significant covariates with *p*‐value >.05 were omitted and the horizontal line at *y* = 0 represents *β* = 0.

At the landscape scale, elk selected for westerly aspects (cosine *β* = −0.03, SE = 0.004; sine *β* = −0.02, SE = 0.004), moderate to steep slopes (*β* = 0.06, SE = 0.004), less canopy cover (*β* = −0.28, SE = 0.005), and lower elevations (*β* = −0.02, SE = 0.004; Figure 2). Elk selected areas closer to cover (*β* = −0.12, SE = 0.005), but farther from county roads (*β* = 0.13, SE = 0.004), highways (*β* = 0.03, SE = 0.005), and wolf territory centers (*β* = 0.39, SE = 0.005; Figure 2). Elk avoided deciduous forests (*β* = −0.99, SE = 0.13), coniferous forests (*β* = −0.38, SE = 0.13), idle grasslands (*β* = −1.14, SE = 0.14), forage grasslands (*β* = −2.22, SE = 0.14), forested wetlands (*β* = −2.37, SE = 0.13), mixed forests (*β* = −2.45, SE = 0.15) and lowland scrub/shrub (*β* = −3.11, SE = 0.13; Figure 3).

### 3.2 Landscape scale (2nd order selection): Seasonal models

During spring, elk selected areas with less canopy cover (*β* = −0.17, SE = 0.008), moderate to steep slopes (*β* = 0.14, SE = 0.007), lower elevations (*β* = −0.04, SE = 0.008) with south westerly aspects (cosine *β* = −0.02, SE = 0.008; sine *β* = −0.05, SE = 0.008; Figure 2). Elk selected areas closer to cover (*β* = −0.14, SE = 0.01) and highways (*β* = −0.07, SE = 0.008), yet farther from county roads (*β* = 0.12, SE = 0.007) and wolf territory centers (*β* = 0.53, SE = 0.009; Figure 2). Elk avoided deciduous forests (*β* = −0.78, SE = 0.28), idle grasslands (*β* = −1.49, SE = 0.30), forested wetlands (*β* = −2.00, SE = 0.29), forage grasslands (*β* = −2.01, SE = 0.29), lowland scrub/shrub (*β* = −2.28, SE = 0.29) and mixed forests (*β* = −2.61, SE = 0.32; Figure 3).

During summer, elk selected southerly aspects (cosine *β* = −0.02, SE = 0.01), higher elevations (*β* = 0.04, SE = 0.01), and less canopy cover (*β* = −0.36, SE = 0.01). Elk selected areas closer to cover (*β* = −0.20, SE = 0.01) but farther from county roads (*β* = 0.06, SE = 0.01), highways (*β* = 0.09, SE = 0.01), and wolf territory centers (*β* = 0.08, SE = 0.01; Figure 2). Elk avoided deciduous forests (*β* = −0.43, SE = 0.21), forage grasslands (*β* = −1.94, SE = 0.22), mixed forests (*β* = −1.99, SE = 0.23), idle grasslands (*β* = −2.35, SE = 0.26), forested wetlands (*β* = −2.4999, SE = 0.21), and lowland scrub/shrub (*β* = −3.2779, SE = 0.23; Figure 3).

During fall, elk selected areas with less canopy cover (*β* = −0.39, SE = 0.008), moderate to steep slopes (*β* = 0.04, SE = 0.008) with south‐westerly aspects (cosine *β* = −0.07, SE = 0.008; sine *β* = −0.02, SE = 0.008; Figure 2). Elk selected areas farther from county roads (*β* = 0.13, SE = 0.008), highways (*β* = 0.14, SE = 0.008), and wolf territory centers (*β* = 0.34, SE = 0.009; Figure 2). Elk avoided coniferous forests (*β* = −0.60, SE = 0.17), deciduous forests (*β* = −1.19, SE = 0.17), idle grasslands (*β* = −1.53, SE = 0.18), forage grasslands (*β* = −2.10, SE = 0.17), mixed forests (*β* = −2.64, SE = 0.26), forested wetlands (*β* = −3.15, SE = 0.17), and lowland scrub/shrub (*β* = −3.73, SE = 0.18; Figure 3).

During winter, elk selected areas with less canopy cover (*β* = −0.25, SE = 0.01), westerly aspects (sine *β* = −0.03, SE = 0.01) with moderate to steep slopes (*β* = 0.08, SE = 0.01). They selected areas closer to cover (*β* = −0.15, SE = 0.01) yet farther from county roads (*β* = 0.18, SE = 0.01) and wolf territory centers (*β* = 0.50, SE = 0.01). Elk used all land cover types in proportion to availability (Figure 3).

### 3.3 Home range scale (3rd order selection): Annual model

At the home range scale, elk selected habitats with moderate to steep slopes (*β* = 0.07, SE = 0.01), higher elevations (*β* = 0.06, SE = 0.01), easterly aspects (sine *β* = −0.03, SE = 0.01), and with less canopy cover (*β* = −0.40, SE = 0.01; Figure 2). Elk selected forage grasslands (*β* = 0.89, SE = 0.40), developed‐low intensity (*β* = 0.87, SE = 0.41), crop rotations (*β* = 1.14, SE = 0.42), and idle grasslands (*β* = 1.30, SE = 0.43) and avoided forested wetlands (*β* = −1.13, SE = 0.37), lowland scrub/shrub (*β* = −1.90, SE = 0.37), emergent/wet meadows (*β* = −2.36, SE = 0.38), open water (*β* = −2.66, SE = 0.37), and floating aquatic herbaceous vegetation (*β* = −4.45, SE = 0.72; Figure 3).

### 3.4 Home range scale (3rd order selection): Seasonal models

During spring, elk selected habitats with moderate to steep slopes (*β* = 0.08, SE = 0.01) at higher elevations (*β* = 0.08, SE = 0.02) with westerly aspects (sine *β* = −0.04, SE = 0.01), and less canopy cover (*β* = −0.28, SE = 0.01; Figure 2). Elk selected habitats farther from county roads (*β* = 0.09, SE = 0.01) and wolf territory centers (*β* = 0.08, SE = 0.01; Figure 2). Elk selected forage grassland (*β* = 1.07, SE = 0.50), idle grassland (*β* = 1.32, SE = 0.55), developed‐low intensity (*β* = 1.46, SE = 0.50), crop rotation (*β* = 2.74, SE = 0.57) and avoided emergent/wet meadows (*β* = −2.08, SE = 0.50), open water (*β* = −2.57, SE = 0.47), and floating aquatic herbaceous vegetation (*β* = −3.86, SE = 1.12; Figure 3).

During summer, elk selected habitats with higher elevations (*β* = 0.08, SE = 0.02), less canopy cover (*β* = −0.33, SE = 0.01; Figure 2), and closer to wolf territory centers (*β* = −0.04, SE = 0.01; Figure 2). Elk avoided deciduous forests (*β* = −0.95, SE = 0.39), coniferous forests (*β* = −1.01, SE = 0.39), mixed forests (*β* = −1.03, SE = 0.43), forested wetlands (*β* = −2.40, SE = 0.39), developed‐high intensity (*β* = −2.52, SE = 0.95), lowland scrub/shrub (*β* = −3.05, SE = 0.3961), open water (*β* = −3.63, SE = 0.40), emergent/wet meadows (*β* = −3.7325, SE = 0.643), and floating aquatic herbaceous vegetation (*β* = −5.21, SE = 1.09; Figure 3).

During fall, elk selected habitats with less canopy cover (*β* = −0.50, SE = 0.01), higher elevations (*β* = 0.20, SE = 0.02; Figure 2), that were farther from county roads (*β* = 0.03, SE = 0.01) and closer to wolf territory centers (*β* = −0.09, SE = 0.01; Figure 2). Elk selected for crop rotation (*β* = 0.90, SE = 0.43), idle grasslands habitats (*β* = 0.98, SE = 0.45), forage grasslands (*β* = 1.43, SE = 0.44), and avoided of forested wetlands (*β* = −1.89, SE = 0.39), lowland scrub/shrub (*β* = −2.47, SE = 0.40), developed‐high intensity (*β* = −2.29, SE = 0.93), emergent/wet meadows (*β* = −2.92, SE = 0.42), open water (*β* = −3.08, SE = 0.39), and floating aquatic herbaceous vegetation (*β* = −5.02, SE = 1.09; Figure 3).

During winter, elk selected habitats with less canopy cover (*β* = −0.37, SE = 0.01), gentler slopes (*β* = −0.06, SE = 0.01), higher elevations (*β* = 0.10, SE = 0.02) and northerly aspects (cosine *β* = 0.02, SE = 0.01) that were farther from county roads (*β* = 0.18, SE = 0.02) and closer to wolf territory center (*β* = −0.08, SE = 0.01; Figure 2). Elk used all land cover types in proportion to availability (Figure 3).

## 4 DISCUSSION

As predicted, elk showed spatiotemporally dependent selection for environmental factors across both spatial scales and seasons (Figures 2 and 3). With respect to the spatial scale, selection or avoidance was exhibited across all environmental variables at the landscape scale (i.e., 2nd order; Johnson, 1980) except for land cover types, which were either used less than or in proportion to availability. At the home range scale (i.e., 3rd order; Johnson, 1980), selection or avoidance was not exhibited across all environmental variables (i.e., distance to county roads, highways, wolf territory centers, and canopy cover were neither selected nor avoided) and elk either used habitats in greater proportion than available or in less proportion. This suggests that elk focused on a wider array of environmental characteristics when establishing their home range on the landscape, particularly focusing on the distance from risky habitat features. However, distance from risky habitat features was not significant when choosing areas within their home range. Instead, elk focused on selecting land cover types such as grasslands, developed‐low intensity areas, as well as crop rotations, while avoiding wetlands, floating aquatic vegetation, wet meadows, open water, and lowland scrub/shrub.

This pattern was less clear when examining selection across seasons at both spatial scales. Interestingly, risky habitat features (e.g., distance to county roads and wolf territory centers) became an important consideration at the home range scale (i.e., 3rd order; Johnson, 1980) when assessing elk use seasonally. Elk selected similar distances (either farther from or closer to these features) across seasons and scales, except for summer and winter, where at the landscape scale, they selected areas farther from wolf territory centers while at the home range scale, they selected areas closer to wolf territory centers. In addition, use of land cover types differed in spring and fall, with elk using certain types (e.g., grasslands, crop rotation, developed‐low intensity) in greater proportion than available at the home range scale compared to using them in proportion to available or less than at the landscape scale (i.e., 2nd order; Johnson, 1980).

Our second prediction, that strength of selection (use relative to availability) for environmental factors would be more strongly correlated to factors that influence survival at the largest spatial scale, was partially supported. At the landscape scale (i.e., 2nd order; Johnson, 1980), elk annually showed stronger selection for habitats closer to ≥40% cover (i.e., escape cover), further from risky habitat features (e.g., wolf territory centers, county roads and highways), and showed use of land cover types either in proportion to or less than what is available on the landscape. While at the home range scale (i.e., 3rd order; Johnson, 1980), elk showed nearly double the strength of selection for habitats closer to 40% cover, used land cover types in greater proportion to what is available, and were not influenced by risky habitat features. However, the strength of selection for these environmental factors varied seasonally across scales with no evident pattern. These results support research conducted early in Wisconsin's elk reintroduction (Anderson, Turner, et al., 2005), indicating a strong behavioral pattern of elk selecting home ranges where their relative risk is reduced (Anderson, Turner, et al., 2005; Mech, 1977).

Early in Wisconsin's reintroduction, elk preferred areas within their home range that were far from wolf territory centers during May–September, which was not supported by our spatial model results (Anderson, Turner, et al., 2005). However, when looking across seasons, we found that distance to wolf territory centers and distance to county roads were significant predictors of elk use, similar to early research (Anderson, Turner, et al., 2005), and directionality of selection depended on season. Our results suggest that elk only selected areas farther from wolf territory centers within their home range during spring, but selected areas closer during summer, fall and winter. By placing their home range in safer habitats (i.e., selecting areas farther from risky features at the landscape scale and closer to escape cover) their overall risk within their home range may be lowered, therefore allowing more variation in selection at the home range scale (i.e., 3rd order; Johnson, 1980) across seasons to maximize temporal differences in resource availability (Merems et al., 2020; Viana et al., 2018). For example, pregnant females need to be more vigilant in late spring during calving (average parturition date in Wisconsin is May 23; Merems, 2023) and early rearing to offset predation risk, which may explain why elk selected for areas further from wolf territory centers in spring (Berg et al., 2021; Lung & Childress, 2007). However, we urge caution against overinterpreting these results, as unique anti‐predator calf‐rearing behavior could have occurred after May 31 and into the summer season. Similarly, elk may select for areas further from wolf territory centers in the fall to offset predation risk during the rutting season, particularly the males (Olson et al., 2019). Again, we urge caution against overinterpretation as we were unable to separate male and female elk in our analyses.

Northern Wisconsin has an extensive network of county roads and highways (Wydeven et al., 2009). Our results affirm previous studies showing elk avoid roadways at larger spatial scales due to increased mortality risk (Anderson, Turner, et al., 2005; Merems, 2023; Rowland et al., 2000). However, during spring elk selected for areas closer to main highways. In the heavily forested landscape of northern Wisconsin, roads act as forest openings and edges, providing earlier forage green‐up (Anderson, Turner, et al., 2005), which is essential during spring as elk recover fat reserves lost over winter while meeting the demands of gestation and lactation (Anouk et al., 2014; Bårdsen & Tveraa, 2012; Monteith et al., 2013; Tollefson et al., 2010). This may suggest that elk are making trade‐offs between forage acquisition and risky habitats during key temporal periods (Merems, 2023). During spring, earlier access to green forage may offset the risk of mortality, but the benefits of this trade‐off likely diminish as forage becomes more available during other seasons.

We found across both spatial scales and all seasons' elk selected habitats with less canopy cover, with selection being more pronounced at the home range scale (i.e., 3rd order; Johnson, 1980). This may suggest that elk are selecting these areas for better access to forage as forest stands in early seral stages have greater nutritional value and forage biomass than closed‐canopy forests (Cook et al., 2016). In the NER, we found a significant difference in nutritional value of digestible energy and biomass between open and early seral habitats and closed‐canopy forests (Merems, 2023). Avoidance of habitats with greater canopy cover became more significant as seasons progressed, with fall being most influential as elk are preparing for winter.

Our findings differ from previous studies which suggest that elk select dense forest stands to take advantage of these energetic benefits of thermal cover (Cook et al., 1998; Lamont et al., 2019) especially during winter (Ewald et al., 2014). Additionally, this can be seen in how elk selected land cover types, both annually and seasonally. Elk avoided dense forest stands both annually and seasonally (excluding winter) at the landscape scale (i.e., 2nd order; Johnson, 1980) and during summer at the home range scale (i.e., 3rd order; Johnson, 1980). Selection for areas with less canopy cover may provide elk with direct solar radiation for warmth, which outweighs the warming effects of long‐wave radiation under the canopy which requires more energy to stay warm (Cook et al., 1998). At the landscape scale, elk selected for a mix of southern and westerly aspects which provide higher solar radiation (D'Eon, 2001) indicating that selection likely provided them with the benefits of thermal energy across all seasons and reduced energetic costs associated with walking through deep snow during winter. However, at the home range scale, elk only selected for westerly aspects during spring which suggests elk are seeking increased solar radiation which may contribute to higher primary production (D'Eon & Serrouya, 2005).

Many methods exist for evaluating the role of scale in habitat selection (Boyce et al., 2003) and we have only addressed the consequences of varying extent for sampling available resources. Our analysis was limited by the frequency of GPS fixes (13 h) and the grain size (30‐m pixels) both restricting our ability to discern selection at short time intervals and fine scales (Laforge et al., 2015). Additionally, we did not have data on the sex of individual elk, preventing us from analyzing and understanding the differences in selection between male and female elk during this study. Although we were unable to understand habitat selection regarding foraging and diet selection due to resolution and available data, we were able to separate our coarse spatial model, temporally, as behavior patterns surrounding foraging decisions can be markedly different across seasons (Apps et al., 2001). Our inferences regarding the extent of suitable habitat and overall resource selection patterns may be contingent on the timing of our study, as the elk population has yet to expand across the entire NER. However, predicting the areas that elk are likely to expand into is greatly improved by understanding current patterns in resource selection.

Our results illustrate how processes at different scales (e.g., physiological tolerances, access to different habitats and forage, physical barriers, etc.) influence decision making by elk (Northrup et al., 2016). This is informative for the continued management of Wisconsin's growing elk population and newly reintroduced eastern elk populations. By projecting predicted elk use across different spatial scales, managers could better identify potential locations of preferred habitats and areas likely to be colonized by expanding populations. Similarly, these projections could provide managers with suggested areas of interest for habitat management (e.g., establish young forest patches) and areas to predict the potential impact of road construction and the development of new roads on elk.

## AUTHOR CONTRIBUTIONS

**Jennifer L. Merems:** Conceptualization (equal); data curation (lead); formal analysis (equal); methodology (equal); validation (lead); visualization (lead); writing – original draft (lead); writing – review and editing (equal). **Anna L. Brose:** Writing – review and editing (equal). **Jennifer Price Tack:** Writing – review and editing (supporting). **Shawn Crimmins:** Conceptualization (equal); methodology (equal); supervision (equal); validation (supporting); writing – review and editing (equal). **Timothy R. Van Deelen:** Supervision (supporting); writing – review and editing (equal).

## CONFLICT OF INTEREST STATEMENT

The authors declare no conflicts of interest.

### OPEN RESEARCH BADGES

This article has earned an Open Data badge for making publicly available the digitally‐shareable data necessary to reproduce the reported results. The data is available at https://doi.org/10.5061/dryad.mpg4f4r51.

## Data Availability

Data are provided from the Dryad Digital Repository. Data for: Scale‐Dependence in Elk Habitat Selection for a Reintroduced Population in Wisconsin, USA. [Dataset]. Dryad. https://doi.org/10.5061/dryad.mpg4f4r51.
